# *EGFR*基因状态与晚期非小细胞肺癌患者一线化疗疗效的关系

**DOI:** 10.3779/j.issn.1009-3419.2015.03.02

**Published:** 2015-03-20

**Authors:** 娜 秦, 权 张, 敬慧 王, 卉 张, 艳斐 顾, 新杰 杨, 曦 李, 嘉林 吕, 羽华 吴, 靖颖 农, 新勇 张, 树才 张

**Affiliations:** 1 101149 北京，首都医科大学附属北京胸科医院，北京市结核病胸部肿瘤研究所 肿瘤内科 Department of Medical Oncology, Beijing Chest Hospital, Capital Medical University, Beijing Tuberculosis and Thoracic Tumor Research Institute, Beijing 101149, China; 2 100015 北京，北京和睦家启望中心 New Hope Cancer Center, United Family Healthcare, Beijing 100015, China

**Keywords:** EGFR, 肺肿瘤, 一线, 化疗, EGFR, Lung neoplasms, First-line, Chemotherapy

## Abstract

**背景与目的:**

表皮生长因子受体（epidermal growth factor receptor, *EGFR*）基因状态是表皮生长因子受体酪氨酸激酶抑制剂（epidermal growth factor receptor tyrosine kinase inhibitor, EGFR-TKI）疗效的预测因素，但其对化疗疗效的预测作用尚不明确。本研究旨在探讨对晚期非小细胞肺癌（non-small cell lung cancer, NSCLC）患者一线化疗疗效的预测意义。

**方法:**

收集首都医科大学附属北京胸科医院自2006年1月10日-2013年12月20日经组织病理学证实的181例Ⅲb期/Ⅳ期的NSCLC患者。分析*EGFR*基因状态、临床特征与化疗疗效及无疾病进展生存期（progression-free survival, PFS）之间的关系。

**结果:**

181例患者均进行了*EGFR*基因检测，*EGFR*突变患者75例（41.4%），野生型为106例（58.6%）。全部患者均接受一线化疗，客观缓解率（objective response rate, ORR）为26.0%，疾病控制率（disease control rate, DCR）为70.2%。*EGFR*突变患者的DCR显著高于*EGFR*野生型患者高（84.0% *vs* 60.4%, *P*=0.001）。亚组分析显示，19外显子缺失突变患者化疗的ORR、DCR均高于*EGFR*野生型患者（*P*值分别为0.049，0.002）。21外显子L858R突变患者的DCR高于*EGFR*野生型患者（*P*=0.010）。全部患者中，168例患者可评价PFS，中位PFS为4.3个月，其中腺癌患者PFS较鳞癌患者延长（4.7个月 *vs* 3.0个月，*P*=0.036）；突变患者PFS长于野生型患者（6.3个月 *vs* 3.0个月，*P*=0.002）；体力状况评分（performance status, PS）0-1分组患者PFS较评分为2分延长（4.4个月 *vs* 0.7个月，*P*=0.016）。*Cox*多因素分析显示，*EGFR*突变是影响PFS的独立因素（HR=0.654, 95%CI: 0.470-0.909, *P*=0.012）。

**结论:**

*EGFR*突变是晚期NSCLC患者一线化疗PFS的预测因素。

## Abstract (en)

**Background and objective:**

Status of epidermal growth factor receptor (*EGFR*) gene is a predictor of response to EGFR tyrosine kinase inhibitor (TKI). However, little is know about the relationship between EGFR status and response to chemotherapy. We evaluated the prediction value of *EGFR* mutation status on response to first-line chemotherapy in patients with advanced non-small-cell lung cancer (NSCLC).

**Methods:**

The data of 181 patients with stage Ⅲb/Ⅳ NSCLC who diagnosed by histopathology from January 10, 2006 to December 20, 2013 in Beijing Chest Hospital, Capital Medical University were collected. The relationships between *EGFR* gene status, clinical characteristics and response and progression-free survival (PFS) were analyzed.

**Results:**

All of the 181 patients' *EGFR* statuses were determined. 75 (41.4%) patients harbored *EGFR*-activating mutations and 106 (58.6%) patients were *EGFR* wild-type. All patients received first-line chemotherapy. The objective response rate (ORR) was 26.0% and disease control rate (DCR) was 70.2%. Patients with *EGFR*-activating mutations had a higher DCR than patients with *EGFR* wild-type (84.0% *vs* 60.4%, *P*=0.001) did. Subgroup analysis showed that the ORR and DCR in patients with *EGFR* exon 19 deletions were remarkably higher than those with *EGFR* wild-type (*P* = 0.049, 0.002, respectively). The DCR in patients with *EGFR* exon 21 L858R mutation was significantly higher than that in patients with *EGFR* wild-type (*P*=0.010). 168 patients were available for response evaluation in all of 181 patients and median PFS was 4.3 mo. The PFS of patients with adenocarcinoma was significantly higher than that patients with squamous cell carcinoma (4.7 mo *vs* 3.0 mo, *P*=0.036). The PFS in patients harbored *EGFR*-activating mutations was significantly higher than that in the patients with *EGFR* wild-type (6.3 mo *vs* 3.0 mo, *P*=0.002). The PFS of patients with a performance status (PS) of 0-1 was significantly higher than that in patients with a PS of 2 (4.4 months *vs*. 0.7 months, *P*= 0.016). *Cox* multivariate analysis indicates the *EGFR*-activating mutation is an independent factor affecting PFS (HR=0.654, 95%CI: 0.470-0.909, *P*=0.012).

**Conclusion:**

*EGFR*-activating mutation is a predictor for PFS of first-line chemotherapy in advanced NSCLC patients.

肺癌发病率和死亡率均居我国恶性肿瘤第一位。2012中国肺癌登记年报统计，我国男性和女性肺癌分别占全部恶性肿瘤发病率的22.14%和14.36%，而死亡率则分别占27.21%和21.91%，30年来死亡率增加了464.84%^[1]^。非小细胞肺癌（non-small cell lung cancer, NSCLC）约占全部肺癌的80%-85%，5年生存率不到15%^[2]^。多数患者就诊时已属于局部晚期或发生远处转移。多项研究证实表皮生长因子受体（epidermal growth factor receptor, *EGFR*）基因状态是表皮生长因子受体酪氨酸激酶抑制剂（epidermal growth factor receptor tyrosine kinase inhibitor, EGFR-TKI）疗效的预测因子^[3, 4]^。此外，化疗仍然是晚期NSCLC的传统治疗，目前对于EGFR基因状态与化疗疗效之间的关系尚未十分明确。

IPASS研究亚组分析中，*EGFR*突变患者的化疗的客观缓解率优于野生型患者（47.3% *vs* 23.5%）^[3]^，而Park等^[5]^研究发现，*EGFR*突变患者一线化疗的有效率与野生型患者无差异（34.1% *vs* 35.0%）。因此我们拟通过回顾性分析181例*EGFR*基因状态已知的Ⅲb期/Ⅳ期的NSCLC一线接受化疗的疗效及无疾病进展生存期（progression-free survival, PFS），探讨*EGFR*基因状态对晚期NSCLC化疗的预测意义。

## 1 资料与方法

### 1.1 入排标准

入选标准：①2006年-2013年在首都医科大学附属北京胸科医院经组织病理学证实的Ⅲb期/Ⅳ期的NSCLC患者[根据美国癌症联合委员会（American Joint Committee on Cancer, AJCC）肺癌分期标准（第7版）]；年龄18岁-75岁；美国东部肿瘤协作组（Eastern Cooperative Oncology Group, ECOG）制定的体力状况评分（performance status, PS）≤2分；②所有患者均进行了治疗前组织标本的*EGFR*基因检测；③患者未接受过全身抗肿瘤治疗；④至少有一个可测量病灶，根据实体瘤的疗效评价标准（Response Evaluation Riteria in Solid Tumors, RECIST）1.1定义的可测量病灶为靶病灶。排除标准：临床资料不完整；既往接受过全身抗肿瘤治疗；合并其他部位肿瘤者。不吸烟定义为一生中吸烟＜100支^[6]^。

### 1.2 病历资料及疗效评价

#### 1.2.1 治疗方案

含长春瑞滨、吉西他滨、紫杉醇、多西他赛、培美曲赛在内的含铂或单药的化疗方案。长春瑞滨25 mg/m^2^，d1，d8，每21天1次：或吉西他滨l.0 g/m^2^-1.25 g/m^2^，d1，d8，每21天1次；或紫杉醇175 mg/m^2^，d1，每21天1次；或多西他赛75 mg/m^2^，d1，每21天1次；或培美曲赛500 mg/m^2^，d1，每21天1次；联合或不联合顺铂75 mg/m^2^，d1，每21天1次；或卡铂曲线下面积（area under the curve, AUC）=5，d1，每21天1次；或奈达铂75 mg/m^2^，d1，每21天1次。

#### 1.2.2 疗效评价

患者每2周期行疗效评价，按照RECIST 1.1评价疗效：完全缓解（complete response, CR）、部分缓解（partial response, PR）、稳定（stable disease, SD）、进展（progression disease，PD）。（CR+PR）/总人数×100%为客观缓解率（objective response rate, ORR），（CR+PR+SD）/总人数×100%为疾病控制率（disease control rate, DCR）。无疾病进展生存期（progression-free survival, PFS）定义为从首次用药时间到疾病进展或任何原因引起死亡的时间。在数据截止时尚未进展或死亡的患者将以最后一次肿瘤评价的日期计算。

### 1.3 统计学方法

采用SPSS 16.0统计软件进行统计分析。*EGFR*基因状态、临床特征与化疗疗效之间的关系采用χ^2^或*Fisher's*检验。PFS采用*Kaplan*-*Meier*生存曲线进行分析，*Log*-*rank*检验组间差异。*Cox*回归进行PFS的多因素分析。*P*＜0.05为差异有统计学意义。

## 2 结果

### 2.1 临床资料

自2006年1月-2013年12月，共181例晚期NSCLC患者符合研究标准，一般临床料。见表 1。中位年龄为56岁（范围21岁-75岁），男性103例（56.9%），女性78例（43.1%）；腺癌154例（85.1%），鳞癌27例（14.9%）；Ⅳ期152例（84.0%），Ⅲb期29例（16.0%）；不吸烟92例（50.8%），吸烟患者89例（49.2%）；PS 0-1分177例（97.8%），4例患者PS评分2分（2.2%）。171例（94.5%）患者接受含铂方案的化疗，其中含紫杉醇的铂二联方案的患者55例（30.4%），含长春瑞滨的铂二联方案的患者16例（8.8%），含吉西他滨的铂二联方案的患者36例（19.9%），含多西他赛的铂二联方案的患者31例（17.1%），含培美曲赛的铂二联方案的患者33例（18.2%）。因年龄、脏器功能及PS评分等原因10例（5.5%）患者接受非铂类的一线单药化疗。

**1:** 181例NSCLC患者一般资料 Clinical characteristics of 181 patients with NSCLC

Clinical characteristics	*n*(%)
Gender	
Male	103 (56.9)
Female	78 (43.1)
Age (yr)	
< 70	177 (97.8)
≥70	4 (2.2)
Histological type	
Adenocarcinoma	154 (85.1)
Squamous	27 (14.9)
Clinical stage	
Ⅲb	29 (16.0)
Ⅳ	152 (84.0)
Smoking history	
Non-smoker	92 (50.8)
Smoker	89 (49.2)
PS score	
0-1	177 (97.8)
2	4 (2.2)
NSCLC: non-small cell lung cancer; PS: performance status.	

### 2.2 *EGFR*基因突变

所有患者均接受*EGFR*基因状态的检测，检测方法包括液相芯片法和蝎形探针扩增阻滞突变系统（amplification refractory mutation system, ARMS）方法。其中*EGFR*基因突变75例（41.4%），野生型106例（58.6%）。在女性、腺癌、不吸烟患者中*EGFR*基因突变率高，而与年龄、PS评分、临床分期无关。75例*EGFR*基因突变患者中，18外显子突变1例（0.6%），19外显子缺失突变37例（20.4%），21外显子L858R突变33例（18.2%）。另有4例患者存在两种突变，1例患者为外显子20 S768I合并21外显子L858R突变，1例患者为21外显子L858R突变合并T790M突变，1例患者为19外显子缺失突变合并21外显子L858R突变，1例患者为21外显子L858R突变合并18外显子突变。

### 2.3 *EGFR*基因突变状态与化疗疗效

#### 2.3.1 *EGFR*基因突变状态与ORR和DCR之间的关系

181例患者均可评价疗效。其中CR 1例，PR 46例，SD 80例，PD 54例，ORR为26.0%，DCR为70.2%。男性患者的ORR较女性患者高（32.0% *vs* 17.9%, *P*=0.032），ORR与年龄、病理类型、临床分期、吸烟状态、PS评分、*EGFR*基因状态及是否铂二联化疗无关。*EGFR*突变型患者的ORR与*EGFR*野生型患者分别为（32.0% *vs* 21.7%, *P*=0.119），但差异无统计学意义。突变患者的DCR高于野生型患者（84.0% *vs* 60.4%, *P*=0.001），DCR与*EGFR*基因状态有关，而与性别、年龄、病理类型、临床分期、吸烟状态、PS评分及是否铂二联化疗无关（表 2）。

**2:** NSCLC患者化疗ORR、DCR与其临床特征的关系 Relationship between ORR, DCR to chemotherapy and clinical characteristics in patients with NSCLC

Clinical characteristic		ORR			DCR	
		*n*(%)	*P*		*n*(%)	*P*
Gender			0.032			0.571
Male	103	33 (32.0)			74 (71.8)	
Female	78	14 (17.9)			53 (67.9)	
Age (yr)			> 0.999^*^			> 0.999^*^
< 70	177	46 (26.0)			124 (70.1)	
≥70	4	1 (25.0)			3 (75.0)	
Histological type			0.344			0.980
Adenocarcinoma	154	38 (24.7)			108 (70.1)	
Squamous	27	9 (33.3)			19 (70.4)	
Clinical stage			0.497			0.550
Ⅲb	29	9 (31.0)			19 (65.5)	
Ⅳ	152	38 (25.0)			108 (71.1)	
Smoking history			0.097			0.614
Non-smoker	92	19 (20.7)			63 (68.5)	
Smoker	89	28 (31.5)			64 (71.9)	
PS score			0.574^*^			0.584^*^
0-1	177	47 (26.6)			125 (70.6)	
2	4	0 (0.0)			2 (50.0)	
EGFR			0.119			0.001
Mutation	75	24 (32.0)			63 (84.0)	
Wild type	106	23 (21.7)			64 (60.4)	
Chemotherapy			> 0.999^*^			> 0.999^*^
Platinum-based combination	171	45 (26.3)			120 (70.2)	
Single agent	10	2 (20.0)			7 (70.0)	
^*^Fisher test. ORR: objective response rate; DCR: disease control rate.						

#### 2.3.2 *EGFR* 19、21外显子敏感突变与野生型患者的化疗疗效

对仅为*EGFR* 19外显子缺失突变，*EGFR* 21外显子L858R突变，*EGFR*野生型的患者进行统计分析。共有173例患者，其中*EGFR* 19外显子缺失突变36例，*EGFR* 21外显子L858R突变33例，*EGFR*野生型患者104例。*EGFR* 19外显子缺失突变患者一线化疗的ORR（38.9% *vs* 22.1%, *P*=0.049）、DCR（88.9% *vs* 60.6%, *P*=0.002）均高于*EGFR*野生型组患者，可见统计学差异。*EGFR* 21外显子L858R突变患者一线化疗的DCR高于*EGFR*野生型患者（84.8% *vs* 60.6%, *P*=0.010)。而ORR较*EGFR*野生型患者无统计学差异（30.3% *vs* 22.1%, *P*=0.338）。*EGFR* 19外显子缺失突变患者与*EGFR* 21外显子L858R突变患者的ORR及DCR均无统计学差异（38.9% *vs* 30.0%, *P*=0.889; 88.9% *vs* 84.8%, *P*=0.454）。

#### 2.3.3 不同EGFR状态下化疗药物的疗效比较

*EGFR*突变型及野生型患者接受含紫杉醇方案化疗为58例，突变组与野生组患者化疗的ORR（9/25, 36.0%; 9/33, 27.3%; *P*=0.477）及DCR（18/25, 72.0%; 23/33, 69.7%; *P*=0.849）差异无统计学意义。接受含长春瑞滨方案化疗为16例，两组患者化疗的ORR（2/6, 33.3%; 2/10, 20.0%; *P*=0.604）差异无统计学意义，突变组DCR高于野生组（6/6, 100.0%; 4/10, 40.0%; *P*=0.034）。接受含培美曲塞化疗为35例，两组患者化疗的ORR（5/12, 41.7%; 6/23, 26.1%; *P*=0.451）及DCR（10/12, 83.3%; 15/23, 65.2%; *P*=0.434）差异无统计学意义。接受含多西他赛方案化疗为36例，两组化疗的ORR（5/13, 38.5%; 3/23, 13.0%; *P*=0.107）无统计学差异，突变组DCR高于野生组（11/13, 84.6%; 11/23, 47.8%; *P*=0.039）。接受含吉西他滨方案化疗为36例，两组人群化疗的ORR（3/19, 15.8%; 3/17, 17.6%; *P*＞0.999）无统计学差异，突变组DCR高于野生组（18/19, 94.7%; 11/17, 64.7%; *P*=0.037）。

### 2.4 EGFR状态与无进展生存时间的关系

截至2014年4月1日，181例患者中163例患者出现疾病进展，5例患者仍在随访中，13例患者失访。168例患者中位PFS为4.3个月，其中腺癌患者PFS较鳞癌患者有所延长（4.7个月 *vs* 3.0个月，*P*=0.036）（图 1）；*EGFR*突变患者PFS较*EGFR*野生型患者延长（6.3个月 *vs* 3.0个月，*P*=0.002）（图 2）；PS评分0-1分患者PFS较PS评分2分患者延长（4.4个月 *vs* 0.7个月，*P*=0.016）（图 3）。而性别、年龄、临床分期、是否铂二联化疗对PFS的影响无统计学意义。*Cox*多因素分析显示，*EGFR*突变是影响PFS的独立因素（HR=0.654, 95%CI: 0.470-0.909, *P*=0.012）（表 3）。

**1:**
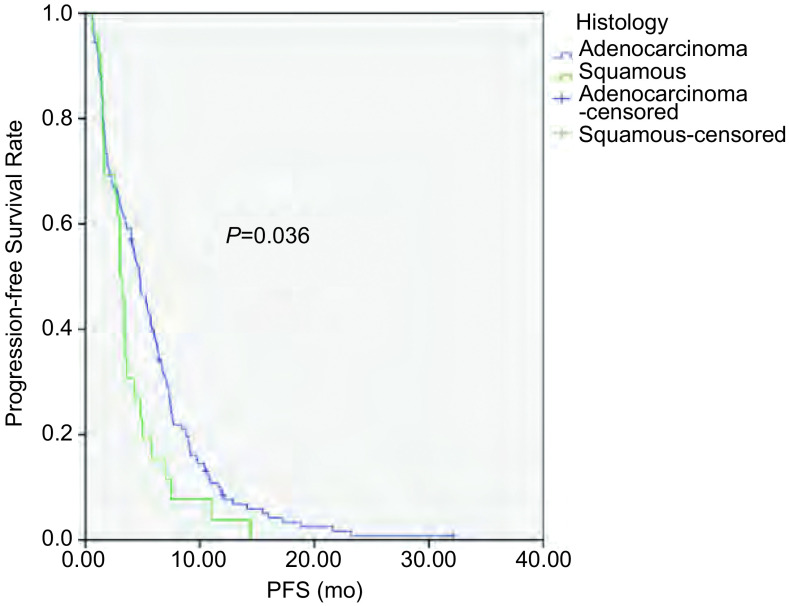
不同病理类型NSCLC患者接受一线化疗的PFS Progression-free survival (PFS) of patients with NSCLC according to histology

**2:**
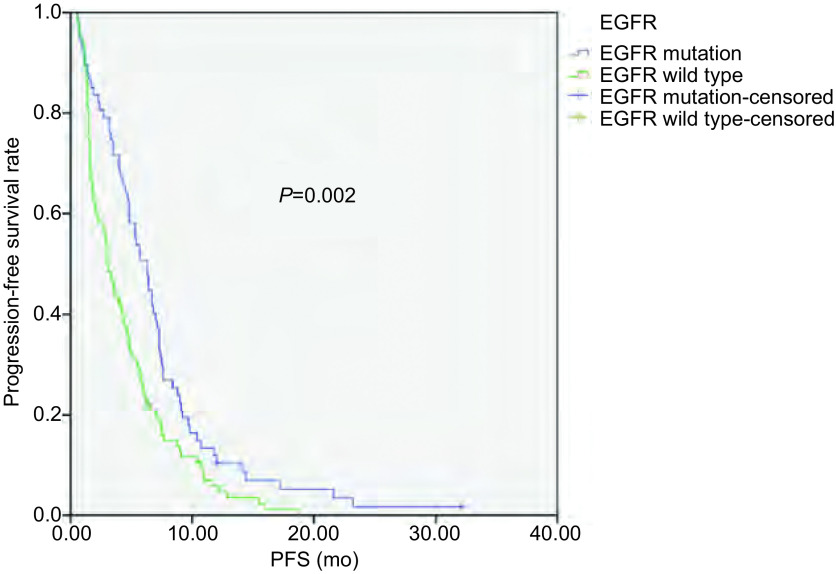
*EGFR*突变与野生型NSCLC患者接受一线化疗的PFS PFS of patients with NSCLC according to *EGFR* mutation status. EGFR: epidermal growth factor receptor.

**3:**
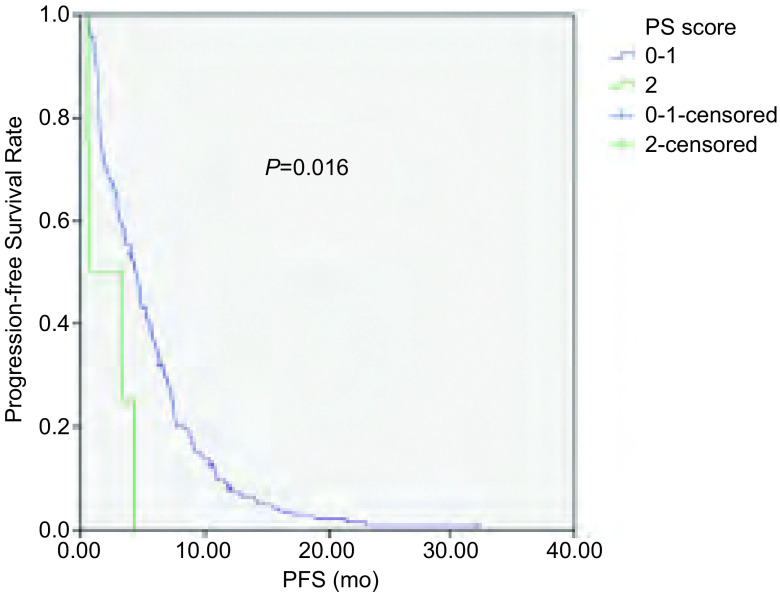
不同PS评分NSCLC患者一线化疗的PFS PFS of patients with NSCLC according to PS score

**3:** 168例NSCLC患者PFS的单因素及多因素分析 Univariate and multivariate analysis of PFS in 168 patients with NSCLC

Clinical characteristic	*n*	PFS (months)		P^*^	Multivariate analysis^△^	
		Median	Range		*P*	HR (95% CI)
Gender						
Male	95	4.3	0.50-18.80	0.334	-	-
Female	73	4.5	0.60-32.10			
Age (yr)						
< 70	164	4.3	0.50-32.10	0.460	-	-
≥70	4	4.8	1.40-12.00			
Histological type						
Adenocarcinoma	142	4.7	0.50-32.10	0.036	0.203	0.753 (0.486-1.166)
Squamous	26	3.0	0.60-14.40			
Clinical stage						
Ⅲb	26	4.8	0.70-32.10	0.688	-	-
Ⅳ	142	4.2	0.50-23.20			
Smoking history						
Non-smoker	86	4.5	0.60-32.10	0.535	-	-
Smoker	82	4.1	0.50-18.80			
PS score						
0-1	164	4.4	0.50-32.10	0.016	0.078	0.401（0.145-1.108）
2	4	0.7	0.60-4.30			
EGFR						
Mutation	67	6.3	0.60-32.10	0.002	0.012	0.654（0.470-0.909）
Wild type	101	3.0	0.50-18.80			
Chemotherapy						
Platinum-based combination	158	4.3	0.50-32.10	0.990	-	-
Single agent	10	3.4	0.60-15.50			
^*^: univariate analysis; ^△^: the variables without significant value in univariate analysis were not errolled in multivariate analysis.						

## 3 讨论

随着分子靶向药物的不断出现及分子分型的普遍开展，靶向治疗为部分晚期NSCLC患者带来明显的疗效，但化疗仍是晚期NSCLC患者的一线标准治疗方案之一。*EGFR*突变是EGFR-TKIs疗效最强的预测因素，但*EGFR*突变状态对化疗疗效的影响结果仍存争议。Gandara等^[7]^研究发现NSCLC患者*EGFR*基因突变与核苷酸切除修复交叉互补基因1（excison repair cross complementation group 1, *ERCC1*）表达水平相关，EGFR基因突变型患者ERCC1低表达。而ERCC1低表达者可能会从以铂类为基础的化疗中获益^[8]^。田玉旺等^[9]^研究也同样发现NSCLC患者中*EGFR*基因野生型与ERCC1高表达有关，*EGFR*基因突变型患者ERCC1低表达者多。由此该研究者推测ERCC1低表达的癌细胞DNA损伤修复能力低，因此*EGFR*基因更易出现突变，对以铂类为基础的化疗敏感。但也有临床研究提示*EGFR*突变会降低多西他赛的敏感性^[10]^。

我们对181例EGFR状态明确的晚期NSCLC患者一线化疗疗效进行回顾性分析，探讨EGFR状态与化疗疗效的关系。结果显示，*EGFR*突变患者的DCR高于*EGFR*野生型患者。19外显子缺失突变患者化疗的ORR、DCR均高于*EGFR*野生型患者，21外显子L858R突变患者的DCR高于*EGFR*野生型患者。*Cox*多因素分析显示，*EGFR*突变是影响PFS的独立因素。

IPASS研究亚组分析中，*EGFR*突变患者的化疗的ORR优于野生型患者（47.3% *vs* 23.5%)^[3]^，Kalikaki等^[11]^报道的162例NSCLC患者中*EGFR*突变组一线接受化疗的有效率明显高于野生型患者（55.6% *vs* 21.8%, *P*=0.023），多因素结果分析显示*EGFR*基因突变是预测化疗疗效的独立因素。Shu等^[12]^报道的266例NSCLC患者，在KRAS阴性的*EGFR*突变组一线接受化疗的有效率高于野生型患者（46.2% *vs* 20.8%, *P*=0.043）。本研究显示*EGFR*突变组患者一线接受化疗的ORR与*EGFR*野生型组的患者无差异（32.0% *vs* 21.7%, *P*=0.119）。分析可能的原因是样本量不是很大，化疗方案未全部统一，其中有10例患者接受了单药化疗。本研究结果提示两组人群在疾病控制率方面具有差异，*EGFR*突变组和野生型组的DCR为（84.0% *vs* 60.4%, *P*=0.001），差异有统计学意义，与尹延涛等^[13]^的研究结果一致。但一项回顾性研究^[14]^结果显示，140例*EGFR*基因状态明确的NSCLC患者一线接受吉西他滨或长春瑞滨联合顺铂或卡铂化疗，*EGFR*基因突变患者与EGFR野生型患者的疾病控制率分别为（74.0% *vs* 82.2%, *P*=0.250），无统计学差异。

亚组分析结果显示*EGFR* 19外显子缺失突变的患者一线接受化疗的ORR及DCR均高于*EGFR*野生型组患者，*P*值分别为0.049、0.002，均有统计学差异。*EGFR* 21外显子L858R突变患者一线化疗的DCR高于EGFR野生型患者，*P*值为0.010，有统计学差异。*EGFR* 19外显子缺失突变患者与*EGFR* 21外显子L858R突变患者的ORR及DCR均无统计学差异。既往对于*EGFR*不同突变类型与化疗疗效的研究较少，Shu等^[12]^的研究结果显示，*EGFR* 19外显子缺失突变患者一线化疗的ORR高于*EGFR* 21外显子突变的患者（37.5% *vs* 24.0%, *P*=0.124）。Cappuzzo等^[15]^的研究结果显示*EGFR* 19外显子缺失突变患者一线接受化疗的ORR优于*EGFR*其他突变类型（46.6% *vs* 0.0%, *P*=0.02），但仅24例，样本量较小，有待大样本的研究进一步证实。

本研究168例患者的中位PFS为4.3个月。单因素分析显示腺癌、PS评分0-1分、*EGFR*基因突变的患者中位PFS更佳，而性别、年龄、临床分期、是否铂二联化疗对PFS的影响无统计学意义。其中*EGFR*突变患者的PFS达6.3个月，长于*EGFR*野生型患者的3.0个月，差异有统计学意义（*P*=0.002）。Eberhard等^[16]^研究结果显示*EGFR*基因突变患者在化疗的ORR及PFS均优于野生型患者（38.0% *vs* 23.0%，*P*=0.01；8.0个月 *vs* 5.0个月，*P*＜0.001）。多因素分析显示*EGFR*突变是PFS的独立影响因素，与其他的研究^[12, 17]^结果一致。而2014年发表的一项的*meta*分析提示晚期NSCLC患者*EGFR*基因突变可能与更高的化疗客观缓解率有关，并没有带来PFS及OS的获益^[18]^。本研究正在对后续治疗及OS进行统计分析以探讨EGFR对晚期NSCLC患者预后的影响。

本研究的不足：本研究为一项回顾性分析，纳入的患者存在选择偏倚，样本量不是很大，*EGFR*基因检测方法及化疗的方案未能统一，这些均有可能对研究的结果产生影响。

本文对181例晚期NSCLC患者一线化疗疗效与EGFR状态的关系进行分析，结果显示*EGFR*突变患者的DCR显著高于*EGFR*野生型患者，*EGFR*突变是影响晚期NSCLC患者一线化疗PFS的独立因素，*EGFR*突变对晚期NSCLC患者一线化疗的PFS具有预测价值，有待于设计良好的前瞻性临床试验进一步明确*EGFR*突变与化疗疗效的关系。
